# Role of the Glyoxalase System in Breast Cancer and Gynecological Cancer-Implications for Therapeutic Intervention: a Review

**DOI:** 10.3389/fonc.2022.857746

**Published:** 2022-07-08

**Authors:** Jingyuan Wang, Xiao Yang, Zhiqi Wang, Jianliu Wang

**Affiliations:** Peking University People’s Hospital, Beijing, China

**Keywords:** glyoxalase system, endometrial cancer, methyglyoxal, ovarian cancer, cervical cancer, breast cancer

## Abstract

Methyglyoxal (MGO), an essential endogenous dicarbonyl metabolite, can lead to multiple physiological problems including hyperglycemia, kidney diseases, malignant tumors, beyond its normal concentration range. The glyoxalase system, making MGO maintained at a low level, links glycation to carcinogenesis, growth, metastasis, and cancer chemotherapy. The glyoxalase system comprises glyoxalase 1 (Glo1) and glyoxalase 2 (Glo2), which is often overexpressed in various tumor tissues. However, very little is known about the glyoxalase system in breast cancer and gynecological cancer. In this review, we introduce the role of the glyoxalase system in breast cancer, endometrial cancer, ovarian cancer and cervical cancer, and highlight the potential of the glyoxalase system to be both as a marker for diagnosis and a novel target for antitumor therapy. However, the intrinsic molecular biology and mechanisms of the glyoxalase system in breast cancer and gynecological cancer need further exploration.

## 1. Introduction

Tumor cells are characterized by uncontrolled growth and proliferation, with an oncometabolic hallmark of favored use of glycolysis for lactate production even under oxygen-rich conditions, referred to as “the Warburg effect” (1–3). This effect is seen as part of metabolic reprogramming in tumors to provide conditions for their proliferation, migration, survival, and drug resistance (3). In fact, the imbalance of energy metabolism is an important driving factor of oncogenesis, with a significant metabolic result being intracellular accumulation of methyglyoxal (MGO). This tends toward causing toxic effects on cells, inhibiting growth and promotion of apoptosis. Increased glyoxalase expression and activity compensate for the accumulation of cytotoxic metabolites in tumor cells. Glyoxalase system, mainly consisting of Glyoxalase 1 (Glo1) and Glyoxalase 2 (Glo2), is a defensive pathway against dicarbonyl stress produced by MGO (4). The formation of MGO increases under conditions of high glycolytic flux, encountered by all cancer cells. When this happens, this detoxification system works and endows tumor cells with adaptive advantage.

Thus, the glyoxalase system is particularly abundant in cancerous cells and this fact has been confirmed by some studies. However, very little is known about the glyoxalase system in gynecological cancer and most work has been done on breast cancer considering female cancers. Thus, the purpose of this review is to introduce the role of the glyoxalase system in breast cancer and gynecological cancer systematically including endometrial cancer, ovarian cancer and cervical cancer, and highlight the potential to be both as a marker for diagnosis and a novel target for antitumor therapy.

## 2. Methyglyoxal (MGO)

MGO which contains two carbonyl groups and is active in nature, together with glyoxal (GO) and 3-deoxyglucosone (3-DG), are referred to as highly reactive dicarbonyl metabolites (5). Among them, MGO is an important endogenous dicarbonyl metabolite that exists in various tissues and organs in the human body, and will cause multiple physiological problems, including hyperglycemia, kidney diseases and malignant tumors, when it exceeds its normal concentration range (6–9). Dicarbonyl stress, which is abnormal increase in the amount of dicarbonyl metabolites, leads to the increase of protein and DNA modification (10). Dicarbonyl stress can be caused by two mechanisms, including out-of-balance of dicarbonyl metabolites and increased exposure of exogenous dicarbonyls (11).

MGO is produced largely by the degradation of glyceraldehyde-3-phosphate (G3P) and dihydroxyacetone phosphate (DHAP) during glycolysis non-enzymatically (4, 12). It can also be produced during hydrolysis and dephosphorylation of DHAP and G3P (13), lipid peroxidation (14, 15), catabolism of threonine (16), oxidation of acetone catalyzed by cytochrome P4502E1 (17), and autoxidation of glucose and degradation of glycated proteins (18, 19). The likelihood of G3P degrading into MGO is eight times than that of DHAP. However, in cells in situ, the concentration of DHAP is about nine times that of G3P (20). Therefore, both forms of triosephosphates are necessary for the formation of MGO (4). MGO is attained not only during cell metabolism, but also through exogenous dietary intake; its primary sources are coffee and other types of beverages (21, 22). However, ingested MGO is metabolized and exerts dicarbonyl stress pre-systemically before absorption (23).

**Figure 1:**
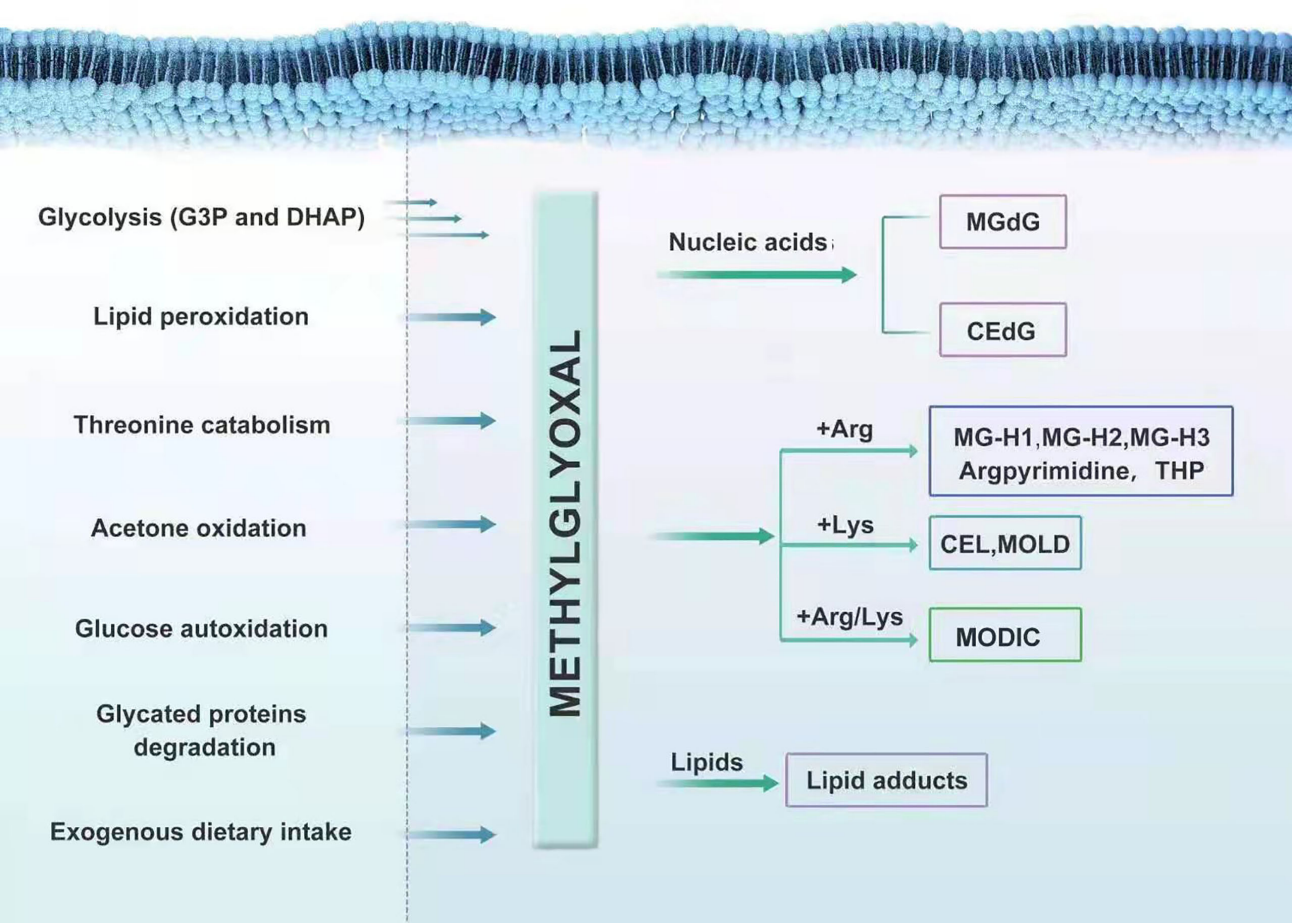
The formation and metabolism of MGO.

MGO is mostly eliminated by the glyoxalase system, with minority being metabolized by aldoketo reductases (AKRs) and aldehyde dehydrogenases (ADHs), which convert it into hydroxyacetone and pyruvate, respectively; thus, forming an enzymatic defense to prevent MGO glycation (24–28). In various human tissues, the capacity of the Glo system to metabolize MGO is 30 times that of AKRs; one exception is the renal medulla, where the expression of AKRs is particularly high (29, 30). In general, MGO is produced during glycolysis and metabolized through the glyoxalase system, at low level *in vivo*. However, when glycolysis is abnormal or food containing high MGO is consumed for a long time, the load of the scavenging system in the body becomes too heavy. This results in the over-accumulation of MGO in the body. The serious cytotoxicity and tissue damage in MGO-related metabolic disorders are likely caused by the modification of nucleic acids, free amino groups in proteins and lipids induced by a large family of MGO-derived adducts, called advanced glycation end-products (AGEs) (31). MGO interacts with deoxyguanosine, leading mainly to form the imidazopurinone adduct, MGdG (26, 32). MGdG, comprising the majority of MGO nucleotide adducts physiologically, are mutagenic and possibly related to malignant transformation (25). A small amount of 2-(1, R/S-carboxyethyl)-deoxyguanosine (CEdG) is also formed (33). The irreversible interaction of MGO with arginine results in the formation of MG-derived hydroimidazolones (MG-H1, MG-H2, and MG-H3) (34–36), argpyrimidine (37) and tetrahydropyrimidine (THP) (38). MGO can also modify lysine residues to form Nϵ-(1-carboxyethyl) lysine (CEL) and 1,3-di(Nϵ-lysino)-4-methyl-imidazolium (MOLD), although to a much lesser extent than arginine (39). MGO can also react with one lysine and one arginine, leading to the formation of an adduct called MODIC (40). MGO also induces stable lipid modifications (**Figure 1**) .

Additionally, AGEs can bind to the receptor for AGEs (RAGE), mediating signal transduction and stimulating intracellular reactive oxygen species (ROS) generation. Activation of RAGE signaling is related to various cellular changes, including inflammation and oxidative stress, which play a role in carcinogenesis, and increased cell death by apoptosis and anoikis (26, 41–44). Decreased expression of RAGE is associated with the inhibition of tumor development and metastasis (45).

## 3. Gyoxal (GO)

In addition to MGO, GO is also an endogenous highly reactive dicarbonyl metabolites. The formation of GO seems to be inevitable in organism, since they are closely connected with several physiological processes, such as lipid peroxidation and degradation of monosaccharides, saccharide derivatives and glycated proteins (46, 47). Since GO is a potent glycating agent, modification of proteins and nucleotides has been found. GO can react with proteins to form AGE residues directly, with arginine residues being the most reactive protein (48). DNA is also susceptible to glycation by GO, with deoxyguanosine (dG) being the most common modified nucleotide AGEs. GO was responsible for increased mutations and decreased DNA replication. Nearly half of mutations were single-base substitutions with more than 80% occurring at C:G sites. Furthermore, GO was in relation to non-random or hotspot mutation sites (49). Similar to MGO, elevation in GO also leads to dicarbonyl stress, which is associated with various health problems and the modification by GO is regarded as damage to physiological systems. However, this can be suppressed by detoxification of GO, catalysed mainly by the glyoxalase system (47, 50). In most cases, GSH was utilized to convert GO to S-2-hydroxyethylglutathione, mediated by GSH-dependent Glyoxalases, Glo1 and Glo2. In some cases, GO can also be the substrate of Glyoxalase 3 (Glo3), but without any cofactors (51, 52).

## 4. Glyoxalase System

**Figure 2:**
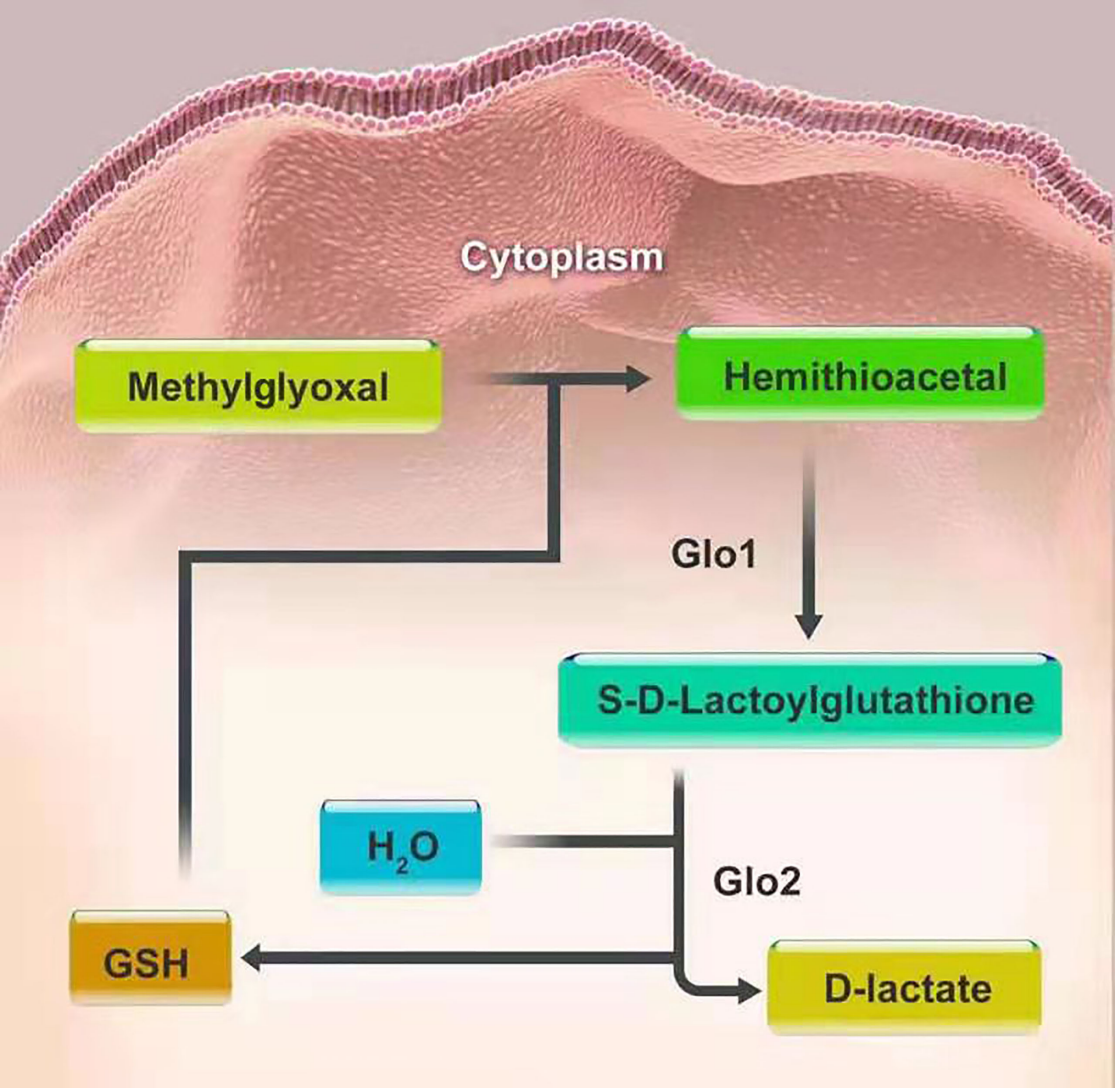
The detoxification of MGO.

The glyoxalase system is one of the well-defined associations between glycation and carcinogenesis and progression. It was first introduced by Dakin, Dudley in 1913 (32). This system, existing in the cytoplasm of all human cells, mainly consists of two cooperating enzymes, namely, Glo1 and Glo2. The main duty of the system is to metabolize MGO and other reactive acyclic a-oxoaldehyde metabolites, to maintain them at a low level, thus preventing cell and tissue dysfunction. MGO and glutathione (GSH) produce hemithioacetal through a non-enzymatic reaction. Then hemithioacetal is converted into S-D-lactoylglutathione, under the catalysis of Glo1. Glo2 catalyzes the hydrolysis of S-D-lactoylglutathione to D-lactate, thereby reforming GSH, to achieve detoxification of MGO (53). In this series of reactions, Glo1, as a rate-limiting enzyme, is of vital importance in the detoxification of MGO (**Figure 2**). There also exists the GSH-independent system involving Glo3 to protect cells from MGO toxicity, besides the GSH-dependent system consisting of Glo1 and Glo2. Glo3 was first identified in the Hsp31 protein from Escherichia coli, which can directly convert MGO into D-lactate in the absence of GSH (54). In recent literature, the DJ-1 proteins from Arabidopsis thaliana and metazoans have also been confirmed to have Glo3 activity like the Hsp31 protein, but they belong to two different subfamilies of the DJ-1 superfamily proteins. In animals, DJ-1 proteins appear to show Glo3 activity and the dysfunction of DJ-1 proteins can make cells sensitive to oxidative stress and cause mitochondrial disorders (54, 55). To date, however, Glo3 remains unidentified in human system. Furthermore, the regulatory mechanisms of Glo3 merit continued study. It is noteworthy that DJ-1 proteins are now considered a deglycase, rather than an alternative glyoxalase (56).

### 4.1 Glo1

Glo1 is a zinc-dependent metalloenzyme encircled by two identical subunits. It has a molecular mass of 43-48 kDa and is comprised of noncovalent bonds (57, 58). It exists in almost all prokaryotic and eukaryotic organisms, including animals, plants, yeast, bacteria, and protozoa. Furthermore, its widespread distribution reflects its important physiological functions in biological life. Moreover, it has been found that Glo1 has high homology among different species through comparison of amino acid sequences. This implies an evolutionarily conserved function for Glo1. Human Glo1 is located on chromosome 6 at locus 6p21.2, with five introns and six exons. It is often overexpressed in tumor tissues (59–61).

The mechanism by which Glo1 expression is regulated is complex. It is controlled by various regulatory elements and that can be altered by the changes in gene expression and post-translational enzymatic modifications (62, 63). Transcriptional regulators of Glo1 include activator protein-2α (AP-2α), early gene 2 factor isoform 4 (E2F4), nuclear transcription factor-κB (NF-κB), activator protein-1 (AP-1), antioxidant response (ARE), metal response (MRE), and insulin response (IRE) elements. Post-translational modifications are achieved through phosphorylation, nitrosylation, and glutathionylation. Among these, Glo1 expression is positively regulated by AP-2α, E2F4, NF-κB and nuclear factor erythroid 2-related factor 2 (Nrf2) by enhancing the Glo1 promoter. For instance, expression is controlled by Nrf2 during stress by binding with AREs (64, 65). In tumor cells, the enhanced expression of Nrf2 leads to increased activity of the enzyme, thereby preventing dicarbonyl stress. Therefore, Nrf2 is often over-activated in hepatocellular carcinoma and thus contributes to increased mortality (66). It has been demonstrated that Nrf2 activators, including sulforaphane and resveratrol, act as Glo1 inducers (67). Nuclear translocation of Nrf2, which is of vital importance in the activation of Glo1, can be disrupted by the combination of MGOs to Kelch-like ECH-associated protein 1 (Keap1). Based on this principle, MGO inhibitors can help detoxification in cells *via* the Nrf2/Keap1 pathway by elevating GSH levels and accelerating MGO metabolism (68). In contrast, the expression of Glo1 is also negatively mediated by hypoxia-inducible factor-1α(HIF1α), the receptor for advanced glycation end products (RAGE). Its levels are also impacted by conflict between the NF-κB system activated in inflammation and Nrf2 (69, 70).

Copy number variation (CNV) of the Glo1 gene in the human genome will also allow for increased expression of Glo1 with low-level duplication in the healthy population (71). The Glo1 DNA segment copy number increase was larger among illness affected groups than that in the healthy population with low-level duplication (72). In a study of 225 different types of human tumors, increased Glo1 copy number was discovered in 8% of tumors, with the highest prevalence of Glo1 amplification in breast cancer (22%), followed by sarcomas (17%) and non-small cell lung cancer (11%) (73). The correlation between Glo1 copy number increase assessed by qPCR and poor survival in gastric cancers has been ameliorated (74).

### 4.2 Glo2

Human Glo2 is another enzyme in the glyoxalase system. It is encoded by the hydroxyacylglutathione gene (HAGH^1^). Genetic polymorphisms of Glo2 are rare, with HAGH^2^ being the only the second phenotype expressed (75). It is a binuclear metalloenzyme with a Zn(II) site as the catalytically active site metal ion. By contrast, the Fe(II) site has no influence on the catalytic activity (70). However, the molecular structures of Glo2 share the same overall fold as Zn(II)-dependent metallo-β-lactamases (76). There are two isoforms of Glo2 depending on their localization in cellular compartments. This includes the mitochondrial form with a molecular mass of 33.8 kDa and a cytosolic form with a molecular mass of 29.2 kDa (77). Despite their differences, the two isoforms have identical isoelectric points of 8.3 (78).

Emerging evidence pointed out the novel independent role of this ancient enzyme from that of Glo1 in a possibly nonenzymatic manner in some malignant cells. In Cinzia et al.’s study, Glo2 was involved in the proapoptotic effects of Oleuropein in non-small-cell lung cancer A549 cells (79). Oleuropein led to an increase in mitochondrial Glo2 protein expression levels without enhancing the enzyme’s activity. Conversely, Oleuropein did not affect expression or function of cytosolic Glo2. Through upregulation of mitochondrial Glo2, Oleuropein is able to induce apoptosis in A549 cells which is mediated by the superoxide anion and Akt signaling pathway. In addition, the proapoptotic effect of mGlo2 is related to the interaction with the Bax protein. Even though there is no distinction between the mitochondrial or cytosolic isoforms, this emerging independent role turns out to be opposite in prostate cancer cells, where an antiapoptotic nonenzymatic role of Glo2 was identified (80). In prostate cancer cells, this ancient enzyme is able to stimulate cell proliferation and elude apoptosis in a mechanism dependent on androgen receptor and involving the p53-p21 axis (81).

## 5. Breast Cancer

Breast cancer is the most common cancer in women worldwide with an estimated 2.09 million cases and 0.6 million deaths annually (82). It is a heterologous disease and categorized corresponding to the so-called intrinsic breast cancer subtypes employing the expression of estrogen receptor (ER), progesterone (PR) and human epidermal growth factor receptor 2 (HER2), together with the frequency of ki-67 (83, 84). Moreover, treatment strategy is designed based on the expression of receptors and ki-67 (5). The use of anti-endocrine therapy to downregulate ER signaling is the primary systemic therapy for ER-positive or PR-positive cases by receptor mediators such as tamoxifen. Patients with HER2-positve benefit from monoclonal antibodies directed against this EGF-receptor, such as trastuzumab and pertuzumab. For triple negative cases, there is no targeted therapy in routine clinical use, thus, most patients are treated with chemotherapy (6). However, this immunohistochemistry-based clinical classification is only a substitute for gene expression analysis and cannot identify all internal molecular characteristics (84).

To our knowledge, the first study giving a comprehensive picture of the role of Glo1 in breast cancer dates back to the 2001, when A Rulli et al. measured Glo1 specific activity in breast carcinoma and normal mammary gland tissue (85). Samples were drawn in the period from 1999 to 2000 of 20 women and Glo1 was significantly upregulated in human breast cancer cells and tissues, as shown by both spectrophotometrical assay and electrophoretic pattern compared with normal counterparts. A consistent enhanced of Glo1 expression was observed either at mRNA or protein level in human breast cancer tissues parallel with pair-matched normal tissue, providing evidence for a potential role of this enzyme in breast cancer (86–89). In addition, Glo1 promoted cell proliferation, invasion and migration and suppressed cell apoptosis. Hence, Glo1 overexpression correlated with aggressive clinicopathological features including lymph node metastasis, lymphovascular invasion, tumor grade and TNM stage and was an independent prognostic factor for clinical outcome of breast cancer patients. Specifically, patients with Glo1 overexpression had a shorter overall survival and recurrence-free survival than those with low Glo1 expression (88, 90). Moreover, knockdown of Glo1 suppressed invasion and migration and promoted apoptosis of breast cancer cells *in vitro (* 88). These results suggest that Glo1 is significantly associated with tumorigenesis, metastasis, and poor prognosis, providing new impetus to the exploring the expression of Glo1. Additionally, gene expression data demonstrated that Glo-1 mRNA was regulated through a mechanism involving inflammation (NF-kB) and oxidative stress (NFE2L2) in malignant breast cells (91). In Guo et al. study, Glo1 restraint treatment can hinder occurrence and development of breast cancer cells, adjusted by actuation of the MAPK signaling pathway and downregulation of Bcl-2 and MMP-9 (92). However, the intrinsic molecular biology and mechanisms of breast carcinogenesis remain to be further elucidated.

Therefore, Glo1 is involved in the regulation of tumorigenesis, proliferation, migration and survival in breast cancer (93). These data has supported the role of Glo1 as a potential target for anticancer drug development, which were indeed confirmed by some studies. Clinically, a major obstacle in the process of treating tumor lies in drug resistance. It has been reported in previous literature that chemotherapeutic resistance, including doxorubicin, was associated with upregulation of Glo1 (94). Recent studies on the mechanism of drug resistance of breast cancer have found that Glo1 inhibitors can reserve drug resistance of tumor cells. Davies et al. showed that the thiazolidinedione troglitazone downregulated Glo1 expression, leading to a regained sensitivity to doxorubicin. Furthermore, it is also reported that Glo1 abundance could predict the outcome of radiotherapy and overexpression of Glo1 was associated with a shorter relapse free survival after receiving radiotherapy (91). It is reported that more than 50% of all drugs used in tumor treatment contain either natural origin active principles or semisynthetic derivatives, thus, there is an urgent need to find new drugs from bioactive compounds (95). In a recent study, the influence of resveratrol, curcumin and piperine on Glo1 activity and expression was assessed in MCF-7 cell. The dose-dependent inhibitory effects of resveratrol, curcumin and piperine on Glo1 activity were observed after 24 hours of treatment. However, the expression of Glo1 could be reduced only by curcumin, due to the possible fact that resveratrol and piperine affect the activity of Glo1 in a posttranslational manner (96). Similar conclusions were also obtained to confirm the effect of curcumin on Glo1 (97, 98).

Moreover, distant metastasis would be present in 15% of patients with breast cancer, and contribute to approximately 90% of cancer-associated mortality (99). Thus, determining potential key regulators in the process of cancer metastasis seems to be increasingly important. According to a recent report, in patients with stage III−IV breast cancer, Glo1 and PKCλ may be cooperatively involved in cancer progression and patients with high Glo1 and PKCλ expression had worse prognosis (87). In addition, the Glo1 inhibitor, TLSC702, and the PKCλ inhibitor, aurothiomalate, may serve as novel pharmacological approaches to manage late−stage breast cancer through suppressing both cell viability and tumor−sphere formation in MDA−MB−157 and MDA−MB−468 human basal−like breast cancer cells. However, there is absence of *in vivo* studies using TLSC702 or aurothiomalate, further investigation of the inhibitors is needed in future.

It is worth noting that in the study of Marie-Julie Nokin et al., a tumor-suppressing role of Glo1 in breast cancer cells was identified for the first time (100, 101). Silencing of Glo1, bearing a higher level of MGO, promoted tumor growth and metastasis *in vivo* and Glo1-depleted breast cancer cells induced a significant increase in pulmonary tumor burden. A similar role of Glo1 has also been validated in hepatocellular carcinoma and downregulation of Glo1 enhanced tumor growth (102). The mechanism was further revealed that metastasis was associated with the activation of MEK/ERK/SMAD1 cascade in breast cancer cells (101). Moreover, this study investigates therapeutic potential of MG scavengers, including carnosine and aminoguanidine, as promising target in the management of metastatic breast cancer.

In fact, these seemingly contradictory data might be explained by the effect exerted by MGO on cancer cells that is defined by low-dose stimulation and high-dose inhibition of tumor metastasis (103). Thus, it is necessary to determine the MGO concentrations when Glo1 inhibitor applied.

## 6. Endometrial Cancer

Endometrial cancer is the most common gynecological malignancy in the United States, and its related mortality is on the rise (104). Surgical staging system including laparoscopic total hysterectomy, bilateral salpingo-oophorectomy, and sentinel lymph-node mapping, has been adopted. Most cases are diagnosed in the early stage of the disease, presenting with vaginal bleeding. Hence, the prognosis is good, with the overall five year survival rate being 90.88% for patients staged as IA using the FIGO 1988 surgical classification (105). However, challenges still remain, including increasing radical disparities in mortality (106).

6.5% of the patients suffered from endometrial cancer are younger than 45 years of age (107). Women of childbearing age prefer to preserve their fertility for future opportunities to give birth, rather than receive the standard surgical treatment of total hysterectomy with bilateral salpingo-oophorectomy, sentinel lymph node mapping and pelvic/para-aortic lymphadenectomy when necessary. Fertility preservation is suitable for young women with stage I, grade I adenocarcinoma. Thus, alternative treatments involving synthetic progestins, including medroxyprogesterone acetate (MPA), are the mainstays of such management. Unfortunately, about 30% of said alternatively treated cases, fail to respond to progestins initially. Although the response rate is approximately 70%, 57% of patients relapse and develop drug resistance (108). In brief, progestin resistance restricts the validity of progestin treatment. Zhang et al. reported that the expression of Glo1 in progestin-resistant Ishikawa cells was increased 2.4-fold higher than that in parental cells. This suggests that Glo1 is related to progestin resistance in endometrial cancer. Further, metformin, an insulin sensitizer, can downregulate Glo1 expression to enhance the response to MPA treatment by blocking PI3K-mTOR activation (109). In another study, metformin sensitizes progestin in endometrial cancer through downregulation of Ten-eleven translocation 1 (TET1), a dioxygenase responsible for transferring 5-methylcytosine into 5-hydroxymethylation and CpG islands enriched in the promoter region of Glo1 are possible target of TET1. Therefore, metformin enhances progestin sensitivity underlying the potential mechanism of TET1/5hmC/GLOI signaling pathway (110). Therefore, the combination of metformin and MPA is likely an effective strategy for conservative treatments of endometrial cancer and accumulating evidence suggests that Glo1 is a potential target gene of metformin.

Traditionally, chemotherapy has been extensively used an adjuvant treatment for endometrial cancer. However, in this case, the initial reaction of malignant endometrial tumor cells to chemotherapy turns refractory over time, resulting in high rates of chemoresistance (111). There is an urgent need to address this issue. Considering that obesity and diabetes are risk factors for the incidence of endometrial cancer, it may be partly caused by metabolic disorders (112). Metformin, a well-tolerated biguanide drug, has been implicated in the treatment of various tumors, including endometrial cancer. According to research, compared with cisplatin and paclitaxel alone, as the first-line chemotherapeutics for endometrial cancer therapy, the administration of metformin strongly inhibits the proliferative activity of tumor cells (113). Further investigation of the possible molecular mechanism by which metformin enhances, chemotherapeutic drug-mediated cytotoxicity, revealed that increasing the dose of metformin reduces the expression of Glo1 protein. This indicates that metformin can enhance sensitivity to chemotherapeutic drugs in endometrial cancer by downregulating Glo1 expression. In fact, since overexpression is present in various cancers, aberrant expression of Glo1 is involved in drug resistance (85, 114). Thus, the expression pattern of Glo1 may play an important role in cancer proliferation.

Previous research has shown that the expression of Glo1 is upregulated in a variety of human malignancies, including melanoma, gastric cancer, pancreatic cancer, breast cancer, renal cancer, prostate cancer (5, 74, 80, 88, 115, 116). This result is similar to that of Sakamoto, who determined that Glo1 enzyme activity was elevated in all 38 human cancer cell lines compared to normal tissue samples (117). High expression of Glo1 is permissive for the survival of tumors with a relatively high flux of MGO formation. Furthermore, elevated Glo1 expression is associated with multidrug resistance in cancer chemotherapy (59). Davies et al. treated doxorubicin-resistant K562 leukemia cells with troglitazone, an insulin sensitizer, and drug resistance was reversed by downregulating the expression of Glo1 (114). The key to inhibiting Glo1 expression to reverse drug resistance lies in promoting cell apoptosis, and there are several potential mechanisms, as described below, although the exact mechanisms are not clear yet. Inhibiting Glo1 expression can result in MGO accumulation to cytotoxic levels that then cause cell death by apoptosis. Therefore, this mechanism is likely caused by increased intracellular MGO, as induced by antitumor agents. On the one hand, MGO has been proven to simulate the release of cytochrome C from mitochondria and subsequently induce apoptosis by modifying the mitochondrial permeability transition pore (118). In addition, nucleic acids and free amino groups in anti-apoptotic proteins can be modified by MGO, thus potentially leading to apoptosis. For example, MGO may enhance the anti-apoptotic activity of Hsp27 by inhibiting the activation of caspase-3 and caspase-9 mediated by cytochrome c to protect cancer tumors from cell deaths (119–121). In this way, compared to cells with low endogenous MGO-modified Hsp27, lung cancer cells with high expression of MGO-modified Hsp27 are resistant to cisplatin-induced apoptosis (119). Similarly, MGO-modified Hsp27 has been found in melanoma, lung, and gastrointestinal tumors (119, 120). Accordingly, Glo1 inhibitors can induce the activation of p38 and JNK stress-activated kinases; which activates downstream caspases in Glo1-overexpressing tumor cells to induce apoptosis (117, 122). Godbout et al. found that the cisplatin-induced apoptosis of myeloma cells was promoted by MGO through activation of protein kinase Cσ (118). Although these results suggest that MGO plays an important role in inhibiting the expression of Glo1, the exact mechanism requires further exploration. According to the literature in the field of endometrial cancer, we found that metformin was an effective inhibitor of Glo1 that had antitumor activity, although the intrinsic mechanism needs to be explored further.

## 7. Ovarian Cancer

Although the incidence of ovarian cancer is not as high as that of other cancers, such as endometrial cancer, it is the most lethal of the female reproductive tract malignancies in the United States (123). Owing to a lack of suitable screening methods, diagnosis is possible only at an advanced stage for most patients; however, at this stage, the tumor has usually spread to the peritoneal cavity and upper abdominal organs, leading to poor prognosis (124). The standard treatment for ovarian cancer focuses on cytoreductive surgery followed by postoperative adjuvant chemotherapy (125). At present, the five-year survival rate is approximately 47% even in countries with advanced medical technology such as the United States and Canada, mainly due to late diagnosis, recurrence, and chemoresistance (126).

Currently, the gold standard for diagnosis relies on pathological biopsy, and early screening methods are limited. Some existing biomarkers such as carbohydrate antigen 125, human epididymis protein 4, may be helpful in screening, but the wide application is hampered by their poor sensitivity or specificity. Thus, it is very necessary to identify novel biomarkers for early detection of ovarian cancer. Considering the fact that blockade of the RAGE-ligand pathway represents a novel target for some cancer therapy (127–130), the researchers have further investigated the role of RAGE in ovarian cancer development. Data showed that RAGE expression was upregulated in ovarian cancer tissue compared with matched normal tissue (131). Moreover, a significant relation between high RAGE expression levels and poor clinicopathological features, such as tumor size, depth of stromal invasion, lymphovascular invasion and stage of tumor was observed, suggesting an important role of RAGE in ovarian cancer progression. In the present study, the area under the curve value was 0.86 for RAGE, implying a relatively high sensitivity and specificity for the RAGE mRNA level to differentiate between malignant and non-malignant tissues. Thus, the overexpression of RAGE may be a potential biomarker for diagnosis of ovarian cancer. Consistent with our results, Poljicanin et al. also came to a similar conclusion (132).

In addition, most ovarian cancers originate from a single layer of surface epithelial cells (OSE), accounting for only a small proportion of the total ovarian mass (133). Apparently, normal OSE cells from women with a family history of ovarian cancer and breast cancer are different from women without phenotypic and/or genotypic family history. Smith Beckerman examined the proteomes of both SV-40-transformed FH-OSE cell lines and control OSE lines. Expression of several proteins appeared to be elevated in the FH-OSE cells, including Glo1, suggesting that high expression of Glo1 is related to the occurrence and progression of ovarian cancer (134). Although ovarian tumors at an early stage are highly curable (135), more than 70% of cases are not diagnosed until the tumor has progressed to advanced stages (136), reflecting the potential high morbidity and mortality caused by presentation with advanced-stage disease. Monica Brown Jones revealed a high degree of overexpression of Glo1 in invasive ovarian cancers compared with the low malignant potential ovarian tumors. Her work combined the technique of laser capture microdissection of epithelial tumor cells in human tissue specimens with two-dimensional gel electrophoresis (137). Results suggest that Glo1 may be a potential marker for early detection and therapeutic targets unique to the invasive phenotype.

The exact mechanisms of Glo1 in the ovarian cancer remain unknown and Glo1 may be used as a therapeutic target in the future. Thus, more investigations are encouraged to provide more reliable data.

## 8. Cervical Cancer

The cause of cervical cancer is clear, being mostly associated with the sexually transmitted persistent human papilloma virus infection. The key to intervention lies in primary and secondary prevention (138). Standard treatment after diagnosis consists of surgical resection and concurrent chemoradiation according to the stage of the tumor and clinicopathologic risk factors. Although the number of cervical cancer cases has decreased in developed countries in the past decade, its incidence has continued to rise rapidly in developing countries (139). According to the latest cancer statistics, cervical cancer is ranked fourth in terms of morbidity and is one of the main causes of death for women with malignant tumors, with approximately 604,000 new confirmed cases as well as 342,000 death cases worldwide in 2020 (140). Thus, cervical cancer still represents a major public health problem globally and there is an urgent need for improved therapeutic options to reduce the burden.

In recent years, more and more researchers have paid attention to phyto chemicals present in various plants, with properties being time tested usage and low toxicity. Hence, Raj Kumar et al. assessed pharmacological action of the inhibitor of Nrf-2, Galangin, an active component of galangal, present in many traditional medicines. Previous reports have confirmed that Galangin contributes to health ranging from antioxidant effect to synergestics anticancer effects with other medicine (141, 142). In the present study, Galangin can modulate Nrf-2 levels to induce cell death and inhibit metastatic potential in human cervical cancer cell line (HeLa) cells *in vitro*. This occurs by downregulating the expression of Glo1 in concentration dependent manner and increasing the damage caused by MGO and oxidative stress (143). In fact, the cytotoxicity of Galangin has been proven in other cancer cell lines, such as human colon cancer cells, melanoma cells, and renal carcinoma cells (144–146). However, little research has been conducted on the role of glyoxalases in cervical cancer. More research is needed along these lines to inform future applications.

## 9. Concluding Remarks

Glyoxalases are often overexpressed in various tumor tissues and they play an important role in tumor proliferation, migration, survival, and drug resistance. In this review, we introduce the role of the glyoxalase system in breast cancer and gynecological cancer, including endometrial cancer, ovarian cancer and cervical cancer. The main function of the glyoxalase system is to metabolize MGO and other reactive acyclic a-oxoaldehyde metabolites, to maintain them at a low level to prevent cell and tissue dysfunction. In most cases, Glo1 overexpression correlated with aggressive clinicopathological features and poor prognosis. However, a tumor-suppressing role of Glo1 has also been identified in breast cancer cells. Due to the possible effect exerted by MGO on cancer cells that is defined by low-dose stimulation and high-dose inhibition of tumor metastasis, it is necessary to determine the MGO concentrations when Glo1 inhibitor applied. These data demonstrated the potential of the glyoxalase system to be as a target for diagnosis and suggested that agents designed to regulate Glo1 may provide a promising method to cancer prevention and therapy. However, the intrinsic molecular biology and mechanisms of the glyoxalase system in breast cancer and gynecological cancer remain to be further elucidated. Therefore, further research is needed in this area.

## Author Contributions

Conceptualization, JYW; methodology, XY; investigation, ZW; writing—original draft preparation, JYW; writing—review and editing, JYW; visualization, ZW; supervision, JLW; funding acquisition, JLW. All authors have read and agreed to the published version of the manuscript.

## Funding

This research was funded by the National Key Technology R&D Program of China (Nos. 2 019YFC1005 200 and 2019YFC1005201), the Natural Science Foundation of Beijing (No. 7202213) and the National Natural Science Foundation of China (No. 82072861, 81672571, and 81874108).

## Conflict of Interest

The authors declare that the research was conducted in the absence of any commercial or financial relationships that could be construed as a potential conflict of interest.

## Publisher’s Note

All claims expressed in this article are solely those of the authors and do not necessarily represent those of their affiliated organizations, or those of the publisher, the editors and the reviewers. Any product that may be evaluated in this article, or claim that may be made by its manufacturer, is not guaranteed or endorsed by the publisher.
